# Surgery of the Duodenum*Published coincidently in Long Island Medical Journal.

**Published:** 1916-01

**Authors:** Edgar R. McGuire

**Affiliations:** Buffalo


### BUFFALO MEDICAL JOURNAL

Yearly Volume 71. JANUARY, 1916. No. 6

### ORIGINAL ARTICLES

The right is reserved to decline papers not dealing with practical medical and surgical subjects, and such as might offend or fail to interest readers. Contributors are solely responsible for opinions, methods of expression and revision of proof.

### *Surgery of the Duodenum

DR. EDGAR R. McGUIRE, Buffalo.

In the developing embryo we have three main divisions of the digestive tract—the foregut, midgut and hindgut. Clinically and anatomically we look upon the pylorus as the dividing line between the stomach and intestines, yet embryologically, we ought to place the dividing line at the junction of the foregut with the midgut, which is where the common bile duct enters the duodenum. In other words, we must consider the first portion of the duodenum as a part of the stomach, rather than as a similar structure to the jejunum. That the duodenum had definite peculiarities of which we know little, is certain. How are we to explain those cases of duodenal dilitation without apparent obstruction? Why should there be such a comparatively high toxicity to obstruction of the duodenum, as compared to any other part of the intestinal tube? Why does ulcer occur so frequently in the first portion of the duodenum, even though so subject to injury, and so constantly exposed to the high acidity of the stomach contents? Again, why should ulcer on one side of the pylorus be so prone to malignant change, and those on the other so resistant to that change? Primary carcinoma of the duodenum is so rare that it is almost a curiosity. And finally, have we an ideal treatment for these lesions?

Originally in the developing embryo the intestinal tube is comparatively straight. The caecum developes, and later rotates across the small intestine, until the normal position of the caecum is reached. Failure of this rotation to take place, explains the abnormal location of the appendix in different in-

*Published coincidently in Long Island Medical Journal.

stances. This normal rotation, however, makes the relation of the duodenum to the greater peritoneal cavity such a difficult matter for the average medical student to understand. Further, the duodenal jejunal junction is now a fixed point, and partial obstruction here, may explain some of the cases of duodenal dilitation. Others, are again explained by failure of the fore-gut and the midgut to accurately unite, thus partially obstructing the lumen. Some of these cases where no obstruction is present, seem to be associated with enteroptosis, these in turn, with intestinal stasis. Possibly the so-called "Lane Kink" and the "Jackson Veils" may be predisposing factors.

You are all familiar with the work of Whipple, Stone, and Bernheim, of Baltimore, on the toxicity of the duodenal mucosa. If a loop of the duodenum be closed (with a short circuit, operation beyond) the animals all die. Injection of the contents of the loop will kill another animal of the same weight. If these loops are drained outside, the animals still die, but if frequent washing of these loops be instituted, the animals will live. It is claimed that this material has the same effect on the other animal, if the bacteria are removed. A similar situation develops in certain cases of duodenal feeding for ulcer. I am informed by some of my medical friends, that it is quite possible to set up a duodenitis which produces most serious symptoms. All of which seems to indicate that under certain forms of obstruction there is some peculiar toxic property to the duodenal mucosa. Murphy, of St. Louis, has also recently reached the conclusion that the toxicity of intestinal obstruction is sometimes due to changes in the mucosa. His further, and very important suggestion lies in the fact that these changes may take place in the mucosa—say in a strangulated hernia—without visible change in the outer coats. Certainly a few cases have died where resection was not made, and autopsy has revealed nothing. This suggests the necessity of possibly removing the mucosa by resection, in delayed cases where the intestinal wall seems to be viable.

In considering the etiology of duodenal lesions, we must view them in their relations to associated conditions. For instance, it would not be fair in considering a breaking down lymph node in the neck, to forget the relation existing between it, and not only the other structures of the neck, but also those inside the mouth. This condition is not unlike infection in the abdomen. We know that the liver is a large organ into which the infected drainage of the entire abdomen is passed, in fact acting as a filter, in a not dissimilar way to the lymph nodes. Doubtless under normal conditions the bile is sterile, but with a primary infected focus in the abdomen, there must come a time when certain infection is carried out in the bile. What role does this play in the production of—first, gall-stones, and second, ulceration of the duodenum? We have heard much of the typhoid organism as a creative factor in cholecystitis, and gall-stones. This is an error, as nearly recent statistics base it below 10%. The very recent work of Rosoneau, shows not only the prevalence of colon bacilli, but also streptococci in cholelithiasis and cholecystitis. Rosonau's work at first thought, would point to the mouth infection as being the only exciting cause of gastric and duodenal ulcer, but on further study it would seem—first, that these are secondary lesions—second, that they are metastatic in character, and third, that the primary lesion may be located almost anywhere. That the tonsils and teeth play the most important role, I do not doubt, but that it is the sole cause is open to question. Recently Dr. E. Gould had a patient, a young girl, taken acutely ill, with patches on her tonsils. The following day she developed pain in the abdomen. Twenty hours later I operated, and found a typical appendix. Cultures taken from the throat showed staphylococci, and scraping from the mucous membrane of the appendix, after washing, showed the same organism. A recent review of this literature, with a report of a similar case, is given by Anderson, in the October issue of the American Journal of the Medical Sciences.

One who has seen much of this work, cannot fail to be impressed by the frequency with which several of these abdominal lesions are co-related. Almost universally one finds a duodenal ulcer, associated with a far from normal appendix. How often in gall-stones, one has been surprised at finding the evidence of old trouble in the appendix. Again, at times, and this seems particularly true of ulcer, one is almost forced to accept Lane's view of ulcer being due to intestinal stasis, because of the frequency with which one finds the two associated. I cannot but feel that the lesions in the upper abdomen are secondary processes, infective in origin, secondary to a primary lesion almost anywhere, but most frequently in the tonsil and infected tooth pockets. Having these theories in mind, the treatment must include attention to the primary lesion in every case. I think the appendix also should be removed in every case of ulcer or cholelithiasis. The treatment of stasis is too uncertain a question for me to venture an opinion, but when present, adequate medical measures at least should be instituted.

Of course one of the vital questions in the treatment of these lesions is—do they undergo malignant change, and if so in what proportion? If malignant changes occur in any considerable proportion of these pyloric ulcers, the treatment must sooner or later be that of excision. On the other hand, if the proportion be small, in what cases are we justified in follow Rodman's suggestion of excision? At present, one side of the problem is championed by the Doctors Mayo, who say that 80% of their cases of cancer of the stomach, show evidence of the malignancy being ingrafted on a chronic ulcer. The other side dispute their claim, even to the extent of denying that the changes spoken of are carcinomatous at all. I am totally unfitted to pass any judgment in this matter, only to add—I have the greatest confidence in the pathological work of the Mayo clinic. Further—the one fundamental fact that seems to be established regarding carcinoma, is that it follows chronic sources of irritation wherever they may be. Until these questions are definitely settled, the matter of excision must be seriously considered in these lesions.

The actual conditions in the duodenum demanding surgery, are congenital obstruction, ulceration with later obstruction or perforation. The treatment is usually a question between some form of excision, and gastro-enterostomy. So much has been written on the subject of gastro-enterostomy for ulcer, that it would seem useless to discuss the matter further. However, we have all found it to be far from an ideal performance in every case. Do not understand me as condemning it entirely, but there are a few objections which must be considered. The early work on gastro-enterostomy gave many instances of biliary regurgitation, but the later short loop, or no loop, has reduced this materially. This is still a serious problem, however. I would refer you to the recent literature where Bloodgood, Erdmann and others, have spoken of its more than occasional occurrence. Erdmann, in a discussion of this subject in the New York Academy of Medicine, said that nearly all of his recent cases of gastro-enterostomy, had required the stomach tube to relieve the dilitation associated with biliary regurgitation. It has happened to me several times within the last two years, once associated with acute dilitation of the stomach, which ended fatally, again, where after repeated washings the patient recovered, and a third, where I made the error of re-opening the abdomen, and found nothing abnormal. It is almost certain where the associated pyloric spasm has been relieved, or the attempted pyloric closure has given way, that the food passes through the pylorus, and not through the anastomosis. I am of the opinion that the excellent results following gastro-enterostomy, occur in those cases where healing of the ulcer caused sufficient contraction to close the pylorus. In any event, **there are many cases where gastro-enterostomy has been performed even in the presence of definite duodenal ulcer, which have failed to be permanently relieved.** Further, surely nature never intended to dispose of the duodenum by any such short circuiting procedure. I am fully in accord with the statement of doctor Finney, when he says

"Gastro-enterostomy ought to be an operation of necessity, never of election."

You will say—if good results follow the closure of the pylorus due to contraction of the ulcer, why not accomplish this in another way. Together with my associates, Drs. Butsch and Evans, I have worked for almost two years, trying out on animals, each of these known procedures for closure of the pylorus. First, a systematic effort was made to occlude the pylorus in normal animals without gastro- enterostomy. This work was reported in the Journal of the American Medical Association, issue of October 3d, 1914. It was done for the purpose of establishing the necessity for or against gastro-enterostomy in cases of acute duodenal perforation. As a result, I can definitely state that it is **impossible to produce obstruction by excising any amount of duodenal wall in normal duodenum.** Even though but a ribbon is left which will admit only the finest probe, it will dilate sufficiently for the ultimate recovery of the animal. Finally I have produced this condition in the human, in about twenty perforations where the ulcer was excised, and no obstruction resulted. I believe it to be impossible to produce obstruction where all of the scar tissue has been excised. If, however, the ulcerating area be not thoroughly excised, obstruction does occur in a considerable proportion of cases. This would seem to prove the inadvisability of performing gastro-enterostomy in the presence of acute, perforations of the anterior wall of the duodenum, where excision is impossible.

The second group of experiments was performed in order to produce pyloric occlusion in the presence of gastro-enterostomy. These may be summarized about as follows: Circular ligatures of catgut and silk, whether tied loosely or tightly, almost invariably pass into the lumen, without producing any permanent obstruction. Purse string sutures of all sorts produce the same result. Wrapping the pylorus with wire—as suggested by Ochsner—strips of aluminum suggested by Brewer, failed in a certain percentage of cases. Fascial strips laid around are more successful, but are not universally so. The round ligament of the liver is usually permanent, because it is a living graft, but again, this is not absolutely trustworthy. The many modifications, including excision of the mucous membrane, either duodenal or stomach, are equally uncertain. The complete division with suture of both ends, whether done first by Von Eiselberg or Doyen, is almost universally successful. Doctor Gerster however, has a patient in which this operation was done, and X-ray shows bismuth going through the pylorus. A rather complete review of this subject is given by Doctor Bartlett, of St. Louis, in the Journal of Medical Sciences, issue of May, 1915.

Lantern slides were shown presenting X-ray pictures of the stomach of dogs following each of the above procedures.

In our series of experiments, we found that while it was impossible to secure complete occlusion by any method excepting that of Von Eiselberg, yet in practically all of the methods, in the presence of a gastro-enterostomy, sufficient occlusion took place by adhesions around the duodenum, to send most of the bismuth through the anastomosis. In our work we found that a great many of the dogs did poorly following any method of gastro-enterostomy. For a time all goes well, but finally loss of weight to the point of extreme emaciation takes place, and often times death. It has seemed to us, that the greater the occlusion of the pylorus in dogs, the more rapid the emaciation. Further, a remarkable dilitation of the stomach occurs in many of these animals, and in many of them the X-ray shows bismuth going through the anastomosis—yet—on autopsy, an anastomosis large enough to admit two fingers was present. Of course we know in the human this is not so, because there has been sufficient number of operations after complete closure, to disprove both features. I merely mention these circumstances in dogs, because I have been unable to explain them.

In reviewing this work, I cannot refrain from adding a remark by that exceedingly clear and gifted surgeon, Doctor W. J. Mayo: "That in the presence of real ulcer, any operation of pyloric occlusion is probably unnecessary." This is doubtless the key note to the whole situation, because the contraction of the ulcer in healing, produces the ideal form of pyloric occlusion. However, if one desires to produce a permanent block for any reason, the Von Eiselberg method is the ideal performance. In view of its greater danger, and the high percentage of closures, clinically, by the other methods, it is unwise in my judgment, to attempt it in any but exceptional instances. The ordinary infolding suture has no mortality, and while not successful in all instances, offers a sufficiently reliable method for most cases.

Surgery in this region is sometimes particularly difficult, because it is almost impossible to distinguish between a benign perforation with inflammatory axudate around the perforation, and malignancy. In a recent discussion on this subject, Doctor Rodman made a perfectly plain statement, that this diagnosis is often impossible, either by clinical methods before operation, or by palpation during laparotomy. I would go further, and say at times the diagnosis is almost impossible by the microscope. In the presence of this dilemman, how is one to decide in favor of an anastomosis or excision? If one accepts the assurance of many, that these become malignant in the future, one must lean towards Rodman's excision. In this connection I have had a few experiences which are of interest, bearing on both sides of the question. In one instance I considered the condition malignant, and inoperable, merely doing an anterior anastomosis. Five years later I operated for an acute perforation of an ulcer in the jejunum, at the sight of the anastomosis and found that the original tumor had entirely disappeared. The patient is still alive and well. In this instance I should have performed a posterior anastomosis, had I not made the error of believing this to be an inoperable carcinoma. On the other hand, Mr. W. was treated for four years for ulcer. X-ray pictures taken one year before operation showed contraction, almost obstruction, quantities of bismuth remaining in the stomach after twenty-four hours. One year later I found a large mass, which on section showed positive carcinoma. This patient has since died of recurrence. This case to my mind is positive evidence that a malignancy can occur on an old ulcer base. In view of these facts, it would seem that the ideal procedure were that of excision, in any doubtful case. However, in view of our lack of knowledge regarding the frequency of this malignant change, and the greater mortality from excision, over gastro-enterostomy, it is probably wiser in most cases to be content with gastro-enterostomy.

When one has a chronic ulcer which has not perforated, conditions are of course different. Here I believe the posterior ones all need a gastro-enterostomy, excision being too hazardous. The anterior ones however, can be easily excised. The ideal procedure would be excision, combined with a Finney gastro-enterostomy. This operation is ideal in that it removes the lesion, and restores the intestinal tube to its normal condition. It is unfortunate that the duodenum is often so fixed as to render this operation difficult, if not quite impossible. In the Journal of Medical Sciences, issue of October, 1915, Finney reviews the end results of 100 cases of gastro-enterostomy, and 100 cases of gastro-duodenostomy. His conclusions favoring the Finney gastro-duodenostomy are of interest in this connection.

With reference to gastro-enterostomy, there are a few suggestions I would like to make regarding the technique which have materially improved my results. **First**—the opening of stomach should not be near the pylorus, but at the lowest part of the stomach, as the Hartman operation favors biliary regurgitation. **Second**—the posterior wall of the stomach should be fastened well down in the greater cavity, to prevent regurgitation. **Third**—hemorrhage from the new opening is best controlled by tying the individual vessels, and not trusting to a running suture alone. **Fourth**—in the opinion of Doctor W. J. Mayo, continued sutures of silk or linen slough into the lumen, and are the cause of secondary ulcers, therefore the suture material should all be of catgut, or possibly interrupted sutures of linen in the outer layer. There seems to be a great prejudice against catgut, but with the improvement in sterilization and hardening, it is now easy to secure a catgut as fine as the usual silk suture, which will last for at least two weeks. In most of my recent cases I have used catgut throughout.

In conclusion I would offer the following: (1) One cannot produce obstruction by excision of the normal duodenum. (2) In the presence of gastro-enterostomy, complete division with suture is the only positive method of blocking the pylorus. (3) Clinically probably most methods of blocking are sufficient, especially in the presence of active ulceration of the duodenum. (4) Excision of the ulcer is the ideal procedure in all cases. (5) Gastro-enterostomy while not ideal, is frequently an operation of necessity.

In closing I want to express my appreciation for the help rendered me by Drs. Butsch and Evans, as the operations on animals were all performed by them. Also for the courtesy shown me by the New York State Institution for the Study of Malignant Diseases, and the University of Buffalo, where the experimental work was done. My obligation to Dr. Koenig for his interest in the X-rays and slides, is also great as you have seen.

430 Delaware Avenue.

---

**Recovery After Operation for Cerebellar Cyst.** Langley Porter of San Francisco, Arch. of Paed., Oct., reports the case of a school girl, aged 11, who had never menstruated. Onset gradual three years before, following jaundice. Fatigue, vertical and prefrontal headache, later vertigo, Sept. 12, 1913, entire cerebellum exposed from above lateral sinuses down to and including posterior part of foramen magnum, from mastoid to mastoid. Sept. 19, after negative exploration along tentorium, cerebello-pontine angles and base of cerebrum, trocar introduced into cerebellar lobes, encountering resistance at depth of 1 C. M. in right, not in left. Cyst found, about 4x8 C. M., opened and clear fluid evacuated. Ultimate recovery apparently perfect.

---

**Malignometer.** Friedrich Klein and Chas. H. Walker, N. Y. Med. Monatsschrift, Oct., recur to their test for sarcoma and carcinoma. Equal parts (2-4 C. C. each) of urine and HCl are mixed without artificial heat. About 10 drops of decinormal or centinormal sodine solution is used and the degree of color indicates the presence of malignancy. At least 20% strength of HCl must be used—about double the strength of the official dilute acid.

